# Medical education in Obstetrics and Gynecology: preferences of medical students regarding digital teaching

**DOI:** 10.3389/fmed.2025.1705733

**Published:** 2025-12-05

**Authors:** Christoph Cirkel, Nikolas Tauber, Natalia Krawczyk, Jann Lennard Scharf, Achim Rody, Maggie Banys-Paluchowski

**Affiliations:** 1 Department of Gynecology and Obstetrics, University Hospital Schleswig-Holstein, Campus Lübeck, Lübeck, Germany; 2 Department of Gynecology and Obstetrics, University Hospital Düsseldorf, Düsseldorf, Germany

**Keywords:** medical education, COVID pandemics, digital teaching, micro-learning, podcast, questionnaire

## Abstract

**Background:**

The aim of this survey is to determine students’ preferences of the University Lübeck in Germany regarding various supplementary digital learning opportunities in the field of Gynecology and Obstetrics in order to better address students’ needs and to improve and modernize teaching.

**Methods:**

An online questionnaire was carried out from the Medical Education Team of the Department of Gynecology and Obstetrics at the University Medical Center Schleswig-Holstein, Campus Lübeck among students during the gynecology rotation at the end of summer semester 2023.

**Results:**

A total of 117 students participated in this online questionnaire [32 male (28%) and 84 female (72%) students]. Hybrid lectures (participation either online or in the lecture hall) were preferred by 111 students (96%), whereas only 2 students (1.7%) favored exclusively in-person attendance. Online learning opportunities were rated as highly or very highly valuable by 93 students (80%). Online learning tools were mainly used for exam preparation [108 students (92%)], for targeted deepening of specific topics [82 students (70%)], to catch up on missed lectures [85 students (72%)] and to review or repeat a lecture content [83 students (71%)].

**Conclusion:**

Traditional teaching methods such as practical exercises and “bed-side teaching”/patient contact are still highly valued by medical students, which students wish to see expanded. Additional online learning opportunities such as on-demand lectures are increasingly important in medical education and are very appreciated by students. Findings indicate that lecturers may consider these needs of the new medical student generation.

## Introduction

The SARS-CoV-2 pandemic led to a global crisis in the economy, society, and the healthcare system worldwide. While before the pandemic, the curriculum at medical faculties primarily consisted of in-person teaching, during the pandemic and especially under the suspension of in-person teaching temporarily mandated by national legislation, the question arose about the redesign and repositioning of medical university education (1).

As part of in-person teaching at the University Medical Center Schleswig-Holstein, Campus Lübeck and generally in Germany as well as in many parts of Europe various subject-specific teaching formats are offered: lecturer-centred lectures in auditoriums, problem-oriented learning, small-group seminars, and clinical clerkships. Attendance at lectures is typically optional, while mandatory group seminars aim to convey course content through interactive discussions and hands-on practice. Further, bedside teaching and clinical clerkships provide students with practical insights. Students’ learning progress is assessed through written examinations and practical tests, including the Objective Structured Clinical Examination (OSCE), a standardized assessment format widely used across Europe to evaluate clinical and communication skills (2).

Within the teaching context of the University Medical Center Schleswig-Holstein, Campus Lübeck, a survey was conducted to evaluate student preferences regarding online and in-person lecture formats and various supplementary digital learning options in the field of Gynecology and Obstetrics. These should serve as supplements in preparing for examinations and clinical clerkships.

Although digital teaching formats were widely implemented during the pandemic, there is still limited evidence on how students in specific clinical disciplines—such as Gynecology and Obstetrics—perceive and evaluate these formats once regular in-person teaching has resumed. This study therefore aims to address this gap by analyzing students’ preferences regarding different teaching modalities within this subject area. The underlying hypothesis is that students’ preferences depend on the type and objectives of the learning activity, with digital formats being particularly valued for flexibility and exam preparation. The findings of this study are intended to contribute to a better understanding of how blended and digital learning components can be meaningfully integrated into the curriculum to support both educational quality and learner satisfaction.

## Materials and methods

The following provides an overview of the questionnaire and the survey.

Participation was entirely anonymous. Multiple responses from the same participant were excluded through IP address verification. By participating in the online survey, participants provided informed consent for their involvement in the study as well as for the anonymous publication of the resulting data. The analysis included only questionnaires from participants who fully completed the survey.

Between May 12, 2023 and July 20, 2023, the Medical Education Team of the Department of Gynecology and Obstetrics at the University Medical Center Schleswig-Holstein, Campus Lübeck carried out an online survey among medical students in their fourth or fifth year of study (out of a total regular study period of approximately 6.5 years in Germany). In addition, midwifery and physiotherapy students were also invited to participate in the survey. Target groups were students participating in the learning course Gynecology and Obstetrics. The questionnaire was administered in German. A standardized digital questionnaire consisting of 19 questions (Supplementary Table 1) was constructed using an online survey platform.^1^ Participants were invited by email, via the official gynecology course homepage and by a reminder during the Gynecology and Obstetrics lecture. Individual questions could be skipped if participants were unable or unwilling to answer them.

The questionnaire was divided into two main sections: (1) student’s interests and teaching preferences and (2) demographic questions. The survey was conducted in accordance with the Office of Academic Affairs of the University of Lübeck. Respondents remained anonymous. Since no personally identifiable information was collected, there was no possibility of revoking the collected data at a later timepoint after completion. Responses to the questionnaire were changeable until final submission. Respondents were informed about the purpose of the survey and actively consented to participate.

Two questions regarded the grade on the *Abitur* (which is important for admission into medical school in Germany and comparable with A-Level grade or Scholastic Aptitude Test or the American College Test) and the grade achieved at the first part of state examination after 2 years of medical studies (so-called *Physikum*). The required average grade of the Abitur for admission to higher education institutions ranges from 1.0 to 4.0 with 1.0 being the best possible grade. To successfully pass the first part of state examinations, the students are required to achieve a grade between 1.0 and 4.0. Grades range from very good (1), good (2), satisfactory (3) to sufficient (4). These variables were collected to obtain a general overview of the participants’ educational background and overall academic performance.

The data analysis was conducted using the common statistical analysis programs Microsoft® Excel® for Microsoft 365 MSO (Version 2509 Build 16.0.19231.20138) and with the Statistical Package for Social Sciences (IBM SPSS Statistics, Version 29.0.2.0, Armonk, NY: IBM Corp.), and the results were evaluated with descriptive statistics. To examine associations between categorical variables, such as the use of online learning resources and students’ age or gender, a chi-square test of independence was applied. This method is particularly suitable for comparing frequency distributions in contingency tables and allows assessment of whether observed group differences are random or statistically significant. The chi-square test was therefore considered the most appropriate approach, as the focus was on descriptive group comparisons rather than on modeling multivariate relationships, as would be the case with more complex methods such as logistic regression. *p* values <0.05 were considered statistically significant. All reported *p* values are two-sided. For the question: “How do you overall assess your personal learning gain from online learning options?,” a mean value analysis was also carried out using a *t*-test for 2 independent variables with two-sided significance. As the Levene test rejected the null hypothesis of equality of variance (significance 0.010), the calculation for sex was based on the assumption of inequality of variance. In the calculation for the age groups, the Levene test confirmed the null hypothesis, so that variance homogeneity was assumed here.

## Results

### Characteristics of the participants

A total of 358 students were invited to participate in the study, of whom 117 completed the survey (response rate 33%). Of the 131 students who started the survey, 117 completed it in full. The completion rate was therefore 89%. Within this group, for whom general descriptive information was available (*n* = 116), 110 students (95%) were studying human medicine, and 6 students (5%) were studying university-level midwifery out of a total of 69 midwifery students among the 358 students invited (8.6%).

In total, 84 students (72%) were female, and 32 students (28%) were male, no sex was stated by 1 student. On average, the respondents were 25 years old (*n* = 116) (range: 21–34), in the 9th university semester or 8th semester in their curriculum (*n* = 114), with a high school grade point average of 1.5 (range: 1.0–3.3) (*n* = 111), and an average score of 2.4 in the first part of their state examination (range: 1.0–4.0) (*n* = 101). A total of 42 respondents (36%) out of 114 reported having completed a previous vocational training: 14 students (12%) in nursing, 6 students (5%) bachelor/master in another university program, and 22 students (19%) in other medical training professions.

### Evaluation and implementation of online learning opportunities

**Figure 1:**
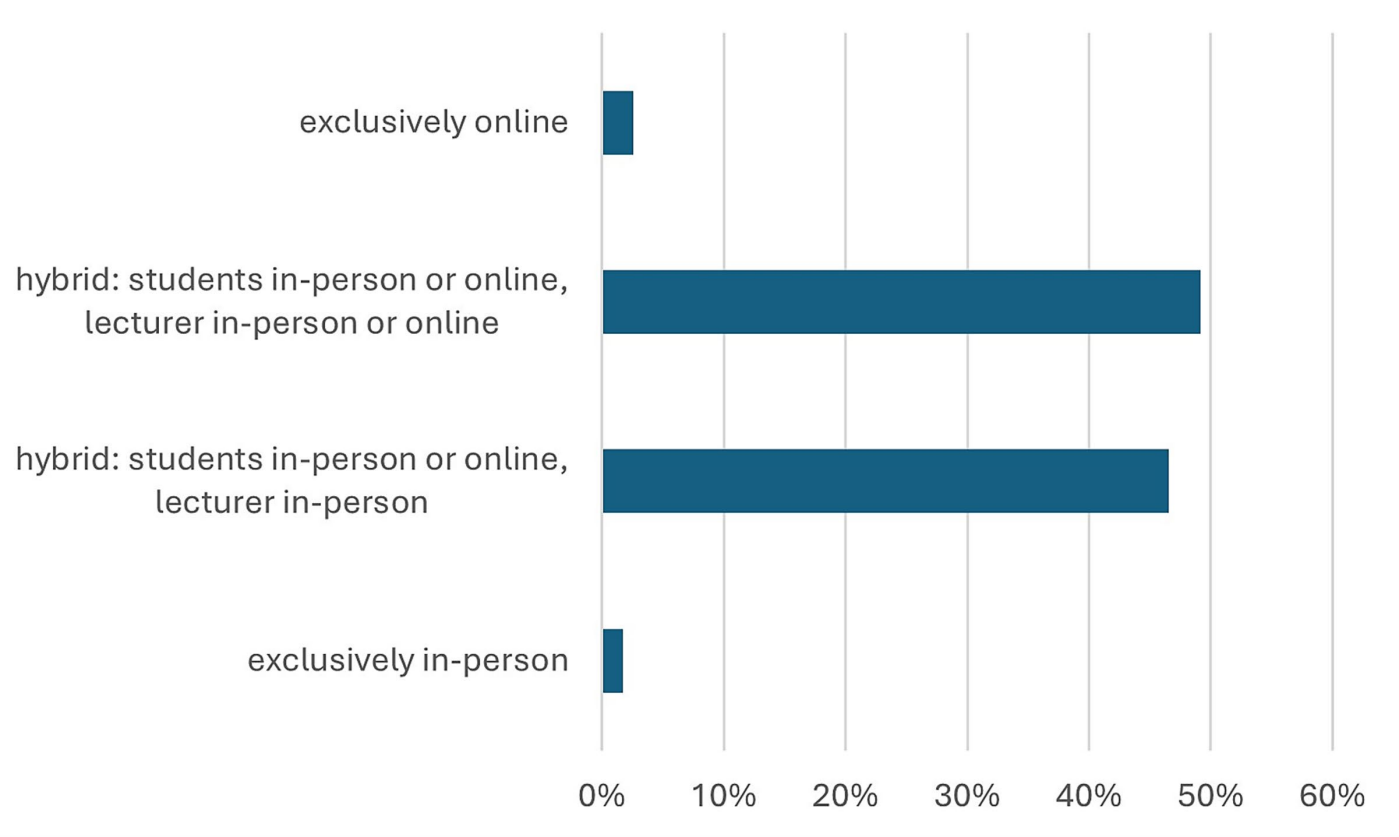
Students’ preferences regarding the mode of lectures.

Medical specialties respondents (*n* = 117) were most interested in (multiple answers were possible) Obstetrics and Gynecology (43 students, 37%), internal medicine (40 students, 34%), paediatrics (39 students, 33%), general medicine (31 students, 27%), and anaesthesiology (30 students, 26%). The majority (89 students, 77%) of respondents wished for additional online learning tools. Participants’ preference on the mode of lecture is shown in Figure 1. Hybrid lectures, allowing participation either in person or online, were preferred by most respondents (111 students, 96%). While 57 students (49%) expressed no preference regarding the lecturer’s mode of participation (in person or remotely connected), 54 students (47%) preferred the lecturer to be physically present in the lecture hall. Only two students (2%) preferred exclusively face to face lectures and three students (3%) exclusively online lectures.

The learning gain from online learning tools was generally rated as high (65 students, 56%) or very high (28 students, 4%) (Table 1), with on-demand lectures and seminars being the most preferable options (86 students, 74%), followed by short online courses summarizing the content of lectures and/or seminars. Online tutorials for specific techniques (77 students, 66%), and micro-learning (63 students, 54%) were also considered helpful.

**Table 1:** Current preferences and previous experiences of students regarding online learning and the length of supplementary podcasts and educational videos.

	*n* (%)	*n* (%) human medicine students	Sex			Age		
			Male	Female	*p*-value	21–25 years	26–34 years	*p*-value
Total	117 (100%)^a^	109 (100%)^a^	32 (28%)^b^	84 (72%)	asymptotic significance 2-sided Pearson chi-square	74 (64%)	41 (36%)	asymptotic significance 2-sided Pearson chi-square
How do you overall assess your personal learning gain from online learning options?					0.066			0.621
Very high	28 (24%)	26 (24%)	7 (18%)	21 (25%)		15 (20%)	12 (29%)	
High	65 (56%)	59 (54%)	14 (43%)	50 (60%)		43 (58%)	21 (51%)	
Medium	29 (17%)	20 (18%)	8 (25%)	12 (14%)		14 (19%)	6 (15%)	
Low	4 (3%)	4 (4%)	3 (9%)	1 (1%)		2 (3%)	2 (5%)	
Very low	0 (0%)	0 (0%)	0 (0%)	0 (0%)		0 (0%)	0 (0%)	
Which format would you prefer for online learning tools?								
Podcasts (audio only) on-demand	36 (31%)	35 (32%)	8 (25%)	28 (33%)	0.386	26 (35%)	10 (24%)	0.234
Micro-learning (short videos of 3–5 min length with one learning objective) on-demand	63 (54%)	58 (53%)	13 (41%)	50 (60%)	0.068	45 (61%)	18 (44%)	0.081
Short online courses summarizing the main content of the respective lecture/seminar on-demand	79 (68%)	73 (67%)	18 (56%)	60 (71%)	0.120	51 (69%)	27 (66%)	0.736
Fully recorded lectures/seminars on-demand	86 (74%)	81 (74%)	24 (75%)	61 (73%)	0.796	50 (68%)	34 (83%)	0.075
Learning videos on specific techniques (e.g., surgical knots)	77 (66%)	70 (64%)	20 (63%)	56 (67%)	0.673	43 (58%)	32 (78%)	0.032^c^
Recorded surgeries on-demand	43 (37%)	40 (37%)	13 (41%)	29 (35%)	0.541	21 (28%)	21 (51%)	0.015^c^
Online courses for in-depth exploration of the subject for those interested (content beyond the learning objectives) on-demand	32 (27%)	32 (29%)	10 (31%)	22 (26%)	0.86	13 (18%)	18 (44%)	0.002^c^
Live online courses (not on demand) for in-depth exploration of the subject for those interested (content beyond the learning objectives)	12 (10%)	12 (11%)	7 (22%)	5 (6%)	0.012^c^	8 (11%)	4 (10%)	0.859
Other	0 (0%)	0 (0%)	0 (0%)	0 (0%)		0 (0%)	0 (0%)	
What do you use/would you use the online learning tools for?								
For exam preparation	108 (92%)	100 (92%)	27 (84%)	80 (95%)	0.051	72 (97%)	34 (83%)	0.006^c^
For targeted deepening of a topic	82 (70%)	75 (69%)	20 (62%)	61 (73%)	0.289	51 (69%)	30 (73%)	0.632
To catch up on a missed lecture	85 (72%)	77 (71%)	22 (68%)	62 (74%)	0.586	52 (70%)	32 (78%)	0.368
To review/repeat a lecture	83 (71%)	75 (69%)	22 (68%)	60 (71%)	0.777	50 (68%)	32 (78%)	0.234
To prepare for state examinations	49 (42%)	46 (42%)	15 (47%)	34 (41%)	0.533	29 (39%)	19 (46%)	0.456
To shorten the waiting times at the university between classes	23 (20%)	23 (21%)	5 (16%)	18 (21%)	0.483	15 (20%)	8 (20%)	0.922
Other: free text: due to greater flexibility in commuting when living in another city	1 (1%)	1 (1%)	0 (0%)	1 (1%)	0.535	0 (0%)	1 (2%)	0.177
Do you already use one or more of the following learning tools?								
Podcasts (audio only) on-demand	25 (25%)	25 (23%)	6 (18%)	19 (22%)	0.651	19 (26%)	6 (15%)	0.169
Micro-learning (short videos of 3–5 min length with one learning objective) on-demand	15 (15%)	14 (13%)	3 (9%)	12 (14%)	0.481	11 (15%)	4 (10%)	0.436
Short online courses summarizing the main content of the respective lecture/seminar on-demand	8 (8%)	6 (6%)	5 (6%)	2 (6%)	0.952	6 (8%)	1 (2%)	0.223
Fully recorded lectures/seminars on-demand	89 (88%)	81 (74%)	22 (69%)	66 (79%)	0.269	53 (72%)	34 (83%)	0.176
Learning videos on specific techniques (e.g., surgical knots)	22 (22%)	21 (19%)	8 (25)	13 (16%)	0.234	12 (16%)	9 (22%)	0.446
Recorded surgeries on-demand	9 (9%)	9 (8%)	2 (6%)	7 (8%)	0.708	6 (8%)	3 (7%)	0.880
Online courses for in-depth exploration of the subject for those interested (content beyond the learning objectives) on-demand	7 (7%)	7 (6%)	0 (0%)	7 (8%)	0.092	5 (6%)	2 (4%)	0.687
Live online courses (not on-demand) for in-depth exploration of the subject for those interested (content beyond the learning objectives)	0 (0%)	0 (0%)	0 (0%)	0 (0%)	0.535	0 (0%)	0 (0%)	0.455
Other: free text: none of the above	1 (1%)	1 (1%)	0 (0%)	1 (1%)	0.651	1 (1%)	0 (0%)	
Which length would you prefer for a podcast (audio only)?					0.488			0.796
– 3–5 min	1 (1%)	1 (1%)	0 (0%)	1 (1%)		1 (1%)	0 (0%)	
– 5–10 min	36 (31%)	33 (30%)	8 (25%)	27 (32%)		22 (30%)	13 (32%)	
– 10–15 min	36 (31%)	33 (30%)	14 (43%)	22 (26%)		25 (34%)	11 (27%)	
– 15–20 min	34 (29%)	33 (30%)	7 (22%)	27 (32%)		21 (28%)	13 (32%)	
– I do not like podcasts	9 (8%)	8 (7%)	3 (9%)	6 (7%)		4 (5%)	4 (10%)	
Which length would you prefer for educational videos?					0.503			0.133
– 3–5 min	16 (14%)	15 (14%)	3 (9%)	13 (16%)		13 (18%)	3 (7%)	
– 5–10 min	53 (46%)	49 (45%)	14 (44%)	39 (46%)		34 (46%)	19 (46%)	
– 10–15 min	27 (23%)	24 (22%)	9 (28%)	17 (20%)		18 (24%)	8 (20%)	
– 15–20 min	19 (16%)	19 (17%)	5 (12%)	14 (16%)		7 (10%)	11 (27%)	
– I do not like educational videos	1 (1%)	1 (1%)	1 (3%)	0 (0%)		1 (1%)	0 (0%)	

a Since not all questions were answered by some participants and multiple answers were allowed in some questions, the sum of all answers may differ from 100%.

b One respondent left the question about sex unanswered.

c Significant correlation according to Pearson (*p* < 0.05).

Respondents used online learning tools primarily to prepare for examinations (108 students, 92%), catching up on missed lectures (85 students, 73%), as well as reviewing and deepening on specific topics (83 students, 71%). So far, recorded lectures and on-demand seminars were most frequently used (89 students, 88%), followed by on-demand audio podcasts (25 students, 25%) and instructional videos for specific techniques (22 students, 22%) (Table 1).

**Figure 2:**
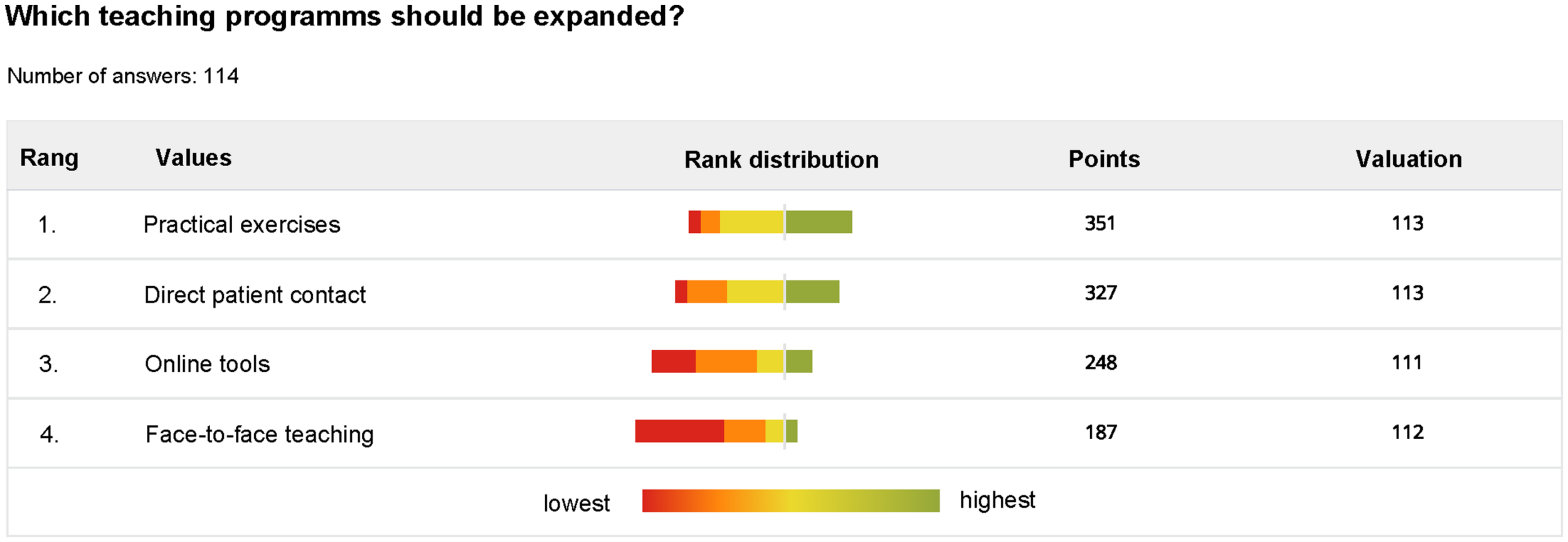
Students’ preferences regarding teaching methods that should be intensified.

The respondents were also asked to rank four teaching methods depending on their preference for further future extension of these teaching formats. Depending on the order chosen by the student, the methods were awarded one to four points. Practical exercises achieved the highest total number (351 points), followed by direct patient contact (327 points) and online tools (248 points). Most students saw the lowest priority in expanding face-to-face teaching (Figure 2).

A further statistical comparison was conducted by dividing the students into two age groups based on the median age of 25 years (Table 1). The first age group (21–25 years) was designated as the younger age group, while the second age group (26–34 years) was designated as the older age group. Interestingly, nearly all students (72, 97%) in the younger age group already use or would use online learning options to prepare for examinations, compared to 34 students (83%) in the older age group (*p* = 0.006), showing a significant difference. In contrast, no significant differences were found with regard to sex or prior vocational training.

The personal learning gain by online learning options was given a mean value of 2.00 (standard deviation SD 0.74) and 1.92 (SD 0.66) for women and 2.2 (SD 0.91) for men in comparison (1 = very high; 2 = high; 3 = medium; 4 = low; 5 = very low). Overall, this indicates only a trend toward a potentially higher benefit for women, but no significant difference between the sexes (*p* = 0.092). There was also no significant difference in the mean values in both age groups (21–25; mean value 2.04 SD 0.71; 26–34 years, mean value 1.95 SD 0.80) although the older age group tended to give slightly higher ratings (*p* = 0.539).

In terms of format preferences, distinct age-specific patterns were observed that may be relevant for designing needs-oriented online curricula. Preferences for learning videos on specific techniques (*p* = 0.032), recorded surgeries (*p* = 0.015), and live online courses for in-depth exploration (*p* = 0.002) were particularly age-dependent: students in the older age group (26–34 years) chose these formats noticeably more often than younger students. Sex-related differences were observed only for the format ‘short online courses,’ which was preferred more frequently by women (*p* = 0.012).

## Discussion

The results of this survey provide new insights into the preferences of students and the opportunities of digital learning tools.

According to the participants of this survey, the implementation of additional online content in medical education is of a high importance. The advantages reported by students are, among others, the increased learning flexibility and focused preparation for examinations. Various studies showed that e-learning technologies are easy to implement and lead to increased satisfaction and motivation for both teachers and students (3–6). As the spectrum of digital learning tools widened, the role of teachers has changed from content deliverers to learning companions and assessors of specific competencies (4). Students benefit from on-demand tools through increased flexibility and individual deepening of learning content based on their priorities and interests. In this context, it is crucial to emphasize that students in our survey, as well as other surveys previously conducted (7–9), generally reject exclusively digital teaching. However, the majority of the students in our survey indicated their wish for hybrid lectures, suggesting a strong preference for deciding their own mode of participation and pace of learning. Interestingly, nearly half of the respondents expect the lecturer to be present in the lecture hall (as opposed to remotely connected). This indicates that the option of face-to-face teaching and direct communication remains an important part of student-teacher interaction.

Hands on teaching especially during clerkships and sub internships in an in- and out-patient setting is more extensive in the German medical curriculum compared to other countries. Particularly the entire final year of their studies consists of mandatory trimester sub internships in Internal Medicine, General Surgery and a subject of choice (10). Before that, they only gain insights into the clinical routine of various disciplines for short periods (freely selectable short internships and defined clinical clerkships), thus acquiring specific practical experiences. Therefore, German medical education offers limited self-determined orientation, making positive experiences in clinical clerkships a major factor that influences the choice of the third elective subject in the final year of medical school. International studies confirm the importance of positive experiences in medical internships and their influence on students’ interest in a particular medical field and the choice of discipline (11–13).

A complete overhaul of the medical curriculum with exclusively digital events and learning tools is not only undesirable for students but also seems counterproductive in terms of acquiring practical skills and positive clinical experiences. Therefore, blended learning concepts, which synergistically combine analogue (clinical clerkships, practical seminars) and digital (lecture recordings, podcasts, micro-learning) learning offerings, have gained particular importance over the last years (7). They allow courses for participants from different training and study programs in the spirit of interprofessional education. Furthermore, they provide options for flexible participation, benefiting students in various life stages (parents, commuters, working professionals etc.) (5, 14–17).

When expanding digital teaching offerings in medical education, several key considerations must be addressed to ensure educational quality and learner outcomes.

Learner engagement is often reduced in online and hybrid formats, with increased distraction and off-task activities documented among online participants, which correlates with lower learning performance compared to on-site learners (18). Strategies to enhance engagement—such as interactive synchronous sessions, regular formative assessments, and structured opportunities for peer and instructor interaction—are essential (19, 20).

Preference for digital formats does not consistently translate into superior assessment scores. While some students report greater satisfaction and flexibility with digital or blended learning, studies show that exam performance may be lower in hybrid or fully digital settings, especially when interactivity and direct communication are limited (18, 21, 22).

In the context of the German medical education system, these findings align with international trends emphasizing the integration of digital tools into traditionally practice-oriented curricula. While universities worldwide have increasingly adopted blended learning models to enhance flexibility and lifelong learning skills (23, 24), the challenge remains to ensure that digital education complements rather than replaces essential clinical experiences (25, 26). At the University Medical Center Schleswig-Holstein, Campus Lübeck, the results of this survey highlight the need to adapt curricular structures in a way that leverages digital tools for preparatory and supplementary learning while preserving direct patient contact and interactive small-group formats as core elements of clinical training. Future curricular reforms should therefore focus on developing a sustainable hybrid teaching framework that supports competence-based learning, fosters self-directed study, and maintains high standards of practical and professional skill acquisition (27, 28). Furthermore, the curricular integration of blended learning formats should be supported by appropriate infrastructural and didactic frameworks, such as faculty development programs, technical platforms, and quality assurance measures.

## Conclusion

In summary, this survey demonstrates the strong preferences of students for the expansion of digital teaching formats. However, it should be noted that a successful expansion of digital teaching in medical education requires robust technical support, active measures to foster engagement, and a blended approach that preserves essential in-person elements to optimize both satisfaction and learning outcomes. Blended learning concepts seem to be a suitable option for combining digital and analog approaches, thereby positively influencing teaching and making it future-oriented.

## Data Availability

The raw data supporting the conclusions of this article will be made available by the authors, without undue reservation.

## Footnotes

1 www.umfrageonline.com
