# Supplementary material for: Medical education in Obstetrics and Gynecology: preferences of medical students regarding digital teaching
Source: Front Med (Lausanne). 2025 Dec 5;12:1705733. doi: 10.3389/fmed.2025.1705733 (PMC12714633; doi:10.3389/fmed.2025.1705733)
Supplement: Supplementary file 1 [file Data_Sheet_1.pdf]

Supplementary Material

Supplementary Table 1. Medical Education Preferences Questionnaire.

	English translation	German original
1.	Which specialty do you like best? (multiple answers possible)<br>- Free text<br>- no preference yet	Welche Fachrichtung gefällt Ihnen am besten? (mehrere Antworten möglich)<br>- Freitext<br>- noch keine Präferenz
2.	Which format do you generally prefer for lectures?<br>- exclusively in person<br>- Hybrid: Students in person or online, lecturer in person or online<br>- Hybrid: Students in person or online, lecturer in person<br>- exclusively online	Welches Format bevorzugen Sie generell bei den Vorlesungen?<br>- Ausschließlich Präsenz<br>- Hybrid: Studierende dürfen online oder in Präsenz teilnehmen, Dozent vor Ort<br>- Hybrid: Studierende dürfen online oder in Präsenz teilnehmen, Dozent vor Ort<br>- ausschließlich online
3.	Would you wish to receive additional online learning opportunities?<br>- yes<br>- no	Wünschen Sie sich ergänzende Online-Lernangebote?<br>- ja<br>- nein
4.	How do you overall assess your personal learning gain from online learning options?<br>- Very high<br>- High<br>- Medium<br>- Low<br>- Very low	Wie beurteilen Sie insgesamt Ihren persönlichen Lerngewinn durch Online-Lernangebote?<br>- Sehr hoch<br>- hoch<br>- mittel<br>- niedrig<br>- sehr niedrig
5.	Which format would you prefer for online learning tools? (multiple answers possible)<br>- Podcasts (audio only) on demand<br>- Micro-learning (short videos of 3-5 minutes length with one learning objective) on demand<br>- Short online courses summarizing the main content of the respective lecture/seminar on demand<br>- Fully recorded lectures/seminars on demand<br>- Learning videos on specific techniques (e.g., surgical knots)<br>- Recorded surgeries on demand<br>- Online courses for in-depth exploration of the subject for those interested (content beyond the learning objectives) on demand<br>- Live online courses (not on demand) for in-depth exploration of the subject for those interested (content beyond the learning objectives)<br>- Other: Free text	Welches Format würden Sie bei Online-Angeboten bevorzugen? (mehrere Antworten möglich)<br>- Podcast (nur Ton, kein Video) on demand<br>- Micro-Learning (kurze Videos von 3-5 Minuten Länge mit jeweils einem Lernziel) on demand<br>- Online-Kurse von 10-15 Minuten mit wichtigsten Inhalten der jeweiligen Vorlesung / des jeweiligen Seminars on demand<br>- Vollständig aufgezeichnete Vorlesungen / Seminare on demand<br>- Lern-Videos zu konkreten Techniken (z.B. chirurgisches Knoten, Ablauf der gynäkologischen Untersuchung, Kolposkopie, Brustbiopsie, Ultraschall…) on demand<br>- Aufgezeichnete Operationen on demand<br>- Online-Kurse zur Vertiefung der Thematik für Interessierte (Inhalt über den Lernzielkatalog hinausgehend) on demand

		- Online-Kurse live (nicht on demand) zur Vertiefung der Thematik für Interessierte (Inhalt über den Lernzielkatalog hinausgehend)<br>- Andere: Freitext
6.	Do you already use one or more of the following learning tools? (multiple answers possible)<br>- Podcasts (audio only) on demand<br>- Micro-learning (short videos of 3-5 minutes length with one learning objective) on demand<br>- Short online courses summarizing the main content of the respective lecture/seminar on demand<br>- Fully recorded lectures/seminars on demand<br>- Learning videos on specific techniques (e.g., surgical knots)<br>- Recorded surgeries on demand<br>- Online courses for in-depth exploration of the subject for those interested (content beyond the learning objectives) on demand<br>- Live online courses (not on demand) for in-depth exploration of the subject for those interested (content beyond the learning objectives)<br>- Other: Free text	Nutzen Sie bereits eines oder mehrere der folgenden Lehrangeboten? (mehrere Antworten möglich)<br>- Podcast (nur Ton, kein Video) on demand<br>- Micro-Learning (kurze Videos von 3-5 Minuten Länge mit jeweils einem Lernziel) on demand<br>- Online-Kurse von 10-15 Minuten mit wichtigsten Inhalten der jeweiligen Vorlesung / des jeweiligen Seminars on demand<br>- Vollständig aufgezeichnete Vorlesungen / Seminare on demand<br>- Lern-Videos zu konkreten Techniken (z.B. chirurgisches Knoten, Ablauf der gynäkologischen Untersuchung, Kolposkopie, Brustbiopsie, Ultraschall…) on demand<br>- Aufgezeichnete Operationen on demand<br>- Online-Kurse zur Vertiefung der Thematik für Interessierte (Inhalt über den Lernzielkatalog hinausgehend) on demand<br>- Online-Kurse live (nicht on demand) zur Vertiefung der Thematik für Interessierte (Inhalt über den Lernzielkatalog hinausgehend)<br>- Andere: Freitext
7.	What do you use/would you use the online learning tools for?<br>- For exam preparation<br>- For targeted deepening of a topic<br>- To catch up on a missed lecture<br>- To review/repeat a lecture<br>- To prepare for state examinations<br>- To shorten the waiting times at the university between classes<br>- Other: Freitext	Wofür nutzen Sie/würden Sie die online-Lehrangebote nutzen? (mehrere Antworten möglich)<br>- zur Klausurvorbereitung<br>- zur gezielten Vertiefung eines Themas<br>- um eine verpasste Vorlesung nachzuhören<br>- um eine Vorlesung nachzuarbeiten<br>- zur Staatsexamensvorbereitung<br>- zur Überbrückung von Wartezeiten in der Uni zwischen Lehrveranstaltungen<br>- Andere: Freitext
8.	Which length would you prefer for a podcast (audio only)?<br>- 3-5 minutes<br>- 5-10 minutes<br>- 10-15 minutes<br>- 15-20 minutes<br>- I don’t like podcasts	Welche Länge würden Sie beim Podcast (nur Ton) bevorzugen?<br>- 3-5 Minuten<br>- 10 Minuten<br>- 15-20 Minuten<br>- ich mag keine Podcasts

9.	Which length would you prefer for educational videos?<br>- 3-5 minutes<br>- 5-10 minutes<br>- 10-15 minutes<br>- 15-20 minutes<br>- I don't like educational videos	Welche Länge würden Sie bei den Lern-Videos bevorzugen?<br>- 3-5 Minuten<br>- 10 Minuten<br>- 15-20 Minuten<br>- ich mag keine Lernvideos
10.	Which teaching method should be intensified? (multiple answers possible)<br>- face-to-face teaching<br>- direct patient contact<br>- practical exercises<br>- online options	Welche Lehrangebote sollten ausgebaut werden? (mehrere Antworten möglich)<br>- Präsenzlehre<br>- Patientenkontakt<br>- praktische Übungen<br>- Online-Angebote
11.	Gender<br>- female<br>- male<br>- diverse	Geschlecht:<br>- weiblich<br>- männlich<br>- divers
12.	How old are you?	Wie alt sind Sie?
13.	Which major are you studying?<br>- Human medicine<br>- Midwifery sciences<br>- Nursing sciences<br>- Physiotherapy	Welchen Studiengang absolvieren Sie?<br>- Humanmedizin<br>- Hebammenwissenschaften<br>- Pflegewissenschaften<br>- Physiotherapie
14.	Which university semester are you enrolled in?	In welchem Hochschulsemester befinden Sie sich?
15.	Which semester of the curriculum are you enrolled in?	In welchem Lehrplansemester befinden Sie sich?
16.	Which high school grade point average *(so-called Abitur* grade) did you achieve?	Welche Abiturnote hatten Sie?
17.	Which grade did you achieved at the first part of state examination (so-called *Physikum*)?	Welche Physikumsnote (1. Abschnitt der ärztlichen Prüfung) hatten Sie?
18.	Have you completed vocational training?<br>- No<br>- Nurse<br>- Paramedic<br>- Physiotherapist<br>- Occupational therapist<br>- Surgical assistant<br>- Medical assistant<br>- Completed university degree<br>- Others	Haben Sie eine abgeschlossene Berufsausbildung?<br>- nein<br>- Krankenpfleger/in<br>- Notfallsanitäter/in<br>- Physiotherapeut/in<br>- Ergotherapeutin/in<br>- Operationstechnische/r Assistent/in (OTA)<br>- Medizinische/r Fachangestellte/r (MFA)<br>- abgeschlossenes Hochschulstudium<br>- Andere
19.	Is there anything else you would like to tell us about this topic?	Möchten Sie uns noch etwas zu diesem Thema mitteilen?
